# *Leishmania* (*Leishmania*) *infantum* DNA detection in *Nyssomyia neivai* in Vale do Ribeira, Paraná, Brazil

**DOI:** 10.1590/0074-02760230173

**Published:** 2024-02-02

**Authors:** Letícia Cristina Morelli, Daniela de Pita-Pereira, Constança Britto, Thais de Araújo-Pereira, Lucas Alexandre Farias de Souza, Kelly de Oliveira Germano, Andrey José de Andrade, Magda Clara Vieira da Costa-Ribeiro

**Affiliations:** 1 Universidade Federal do Paraná, Programa de Pós-Graduação em Microbiologia, Parasitologia e Patologia, Laboratório de Parasitologia Molecular, Curitiba, PR, Brasil; 2 Fundação Oswaldo Cruz-Fiocruz, Instituto Oswaldo Cruz, Laboratório de Biologia Molecular e Doenças Endêmicas, Rio de Janeiro, RJ, Brasil; 3 Centro Universitário Lusíada, São Paulo, SP, Brasil

**Keywords:** leishmaniasis, sand flies, natural infection

## Abstract

**BACKGROUND:**

The incidence of visceral leishmaniasis (VL) has increased in the Southern region of Brazil in recent years, especially in the State of Paraná. New species have been suggested with potential to act as vector in VL endemic areas.

**OBJECTIVES:**

Identify the *Leishmania* species in sand fly specimens collected from 2016 to 2018 in the municipality of Itaperuçu, Vale do Ribeira, Paraná, Brazil.

**METHODS:**

Light traps were used for collections and for the analysis of sand fly were used the multiplex polymerase chain reaction (PCR) methodology and subsequent sequencing.

**FINDINGS:**

Among the collected specimens, 88.62% were attributed to the species *Nyssomyia neivai*, which were grouped into 176 pools. Three positive pools were detected: two with *Leishmania* (*Viannia*) *braziliensis* and one with *L.* (*Leishmania*) *infantum*. The positivity rate for the parasite was 0.25% based on the presence of at least one infected insect in the pool.

**MAIN CONCLUSIONS:**

The detection of *L. infantum* in *Ny. neivai* draws attention due to its abundance and anthropophily in the State of Paraná. Moreover, this finding is considered as an alert and suggests that the vector competence of *Ny. neivai* and the criteria for its incrimination should be carried out, given its wide distribution in southern of Brazil.

Visceral leishmaniasis (VL), also called kala-azar, has been widely distributed in the Americas. In 2021, 93.5% of VL cases registered in the continent were reported in Brazil^1^. The etiological agent of VL in the Americas is the species *Leishmania* (*Leishmania*) *infantum* and its transmission is attributed to the species *Lutzomyia* (*Lutzomyia*) *longipalpis* and *Lutzomyia* (*Lutzomyia*) *cruzi*. However, in the last two decades, studies have presented evidence suggesting that other species of sand flies can function as vectors of *L*. *infantum*, such as *Migonemyia migonei*,^2^ *Pintomyia fischeri* ^3^ and *Nyssomyia neivai*.^4^ ^,^ ^5^

*Nyssomyia neivai* is the sand fly species that has the widest geographic distribution in southern Brazil^6^ and is considered the main vector of American Tegumentary Leishmaniasis (ATL) in the State of Paraná.^6^ ^,^ ^7^ ^,^ ^8^ ^,^ ^9^ According to Silva et al.,^6^ the species was found in high densities in forest, domicile, and peridomicile areas in Vale do Ribeira, which is an endemic region for ATL located in the states of Paraná and São Paulo, evidencing its wide distribution in the region. *Ny. neivai* has a high capacity for adapting to modified environments where there is anthropic action and has already been found naturally infected by *L. infantum.* ^4^ In recent years, the incidence of VL has increased in the Southern region of Brazil, especially in the State of Paraná, where 25 cases of VL have been diagnosed in the last six years.^10^ The municipality of Foz de Iguaçu has in its sand fly fauna the predominant species *Lu. longipalpis*, and it is where the third detection of the presence of DNA from *L. infantum* in *Ny. neivai* was reported.^11^ This scenario increasingly reinforces the need to study the natural infection of *Ny. neivai* and other species in VL endemic areas, regardless of the presence of *Lu. longipalpis*. Thus, this study used the multiplex polymerase chain reaction (PCR) methodology and sequencing in sand flies captured in the municipality of Itaperuçu, located in Vale do Ribeira, Paraná, to evaluate the natural infection in sand flies.

## MATERIALS AND METHODS

*Collection, packaging and assembly of sand flies* - Collections were carried out using modified CDC light traps and Shannon traps (Shannon, 1939) over two years (from June/2016 to July/2018) in the municipality of Itaperuçu (Figure). In a period of 24 months, the CDC traps were installed in the peridomicile of 10 residences for 12 h a day, for three consecutive days. Shannon traps were placed in a forested area for 3 h once a month. For natural infection analysis, specimens from the period between May 2017 and July 2018 were considered. *Ny. neivai* specimens were captured by the respective traps both in peridomicile and forested areas. The traps were installed following the recommendations of the National Leishmaniasis Control Program of the Brazilian Ministry of Health.

**Figure:**
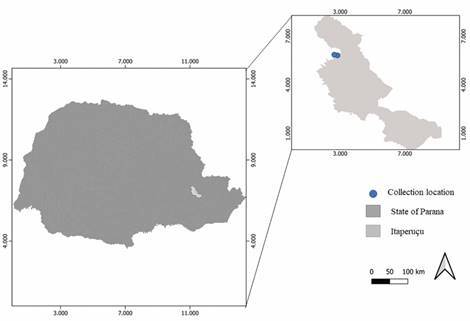
Municipality of Itaperuçu, Vale do Ribeira, Paraná, Brazil.

The captured sand flies were transported to the Laboratory of Molecular Parasitology of the Department of Basic Pathology, Sector of Biological Sciences at the Federal University of Paraná (DPAT/BL/UFPR). They were sorted by external morphology, sexed, and mounted for species-level identification, kept in 80% alcohol, and conserved at -20ºC for extraction and sequencing. The sand flies were identified using the proposal of Galati^12^ and the abbreviation of the genera proposed by Marcondes.^13^

*DNA extraction and multiplex PCR* - A total of 1,186 sand fly specimens were grouped into 176 pools of 2 to 10 specimens each per collection. DNA extraction was performed using the commercial Wizard SV Genomic DNA Purification System kit (PROMEGA^TM^, Madison, WI, USA), following the manufacturer’s specifications.^14^ The Multiplex PCR was designed to simultaneously amplify the cacophony gene in the IVS6 region in sand flies of the neotropical genus *Lutzomyia* (as an internal control for polymerase enzyme activity and DNA extraction) and the conserved minicircle region of DNA from the kinetoplast of *Leishmania* spp. For contamination control, all instruments and work areas were decontaminated with a diluted chloride solution and ultraviolet light.

*Cloning and sequencing* - DNA recovered from each *Leishmania* positive sample was subjected to a second semi-nested-PCR assay targeting the *hsp70* gene. This validated the gene region to distinguish different species of *Leishmania* present in Brazil. In the first round of PCR, a 234 bp fragment of *hsp70* was amplified using the oligonucleotides 5’-GGA CGA GAT CGA GCG CAT GGT-3’ and 5’-TCC TTC GAC GCC TCC TGG TTG-3’. In the second round, the same forward oligonucleotide is paired with the following reverse oligonucleotide: 5’-GGA GAA CTA CGC GTA CTC GAT GAA G-3’ to amplify an internal 144 bp region of the 234 bp fragment. The amplified fragments were purified and cloned into competent *Escherichia coli* DH5α cells using the vector from the pGEM T-Easy Vector kit (Promega^TM^), according to the manufacturer’s recommendations.

Sanger sequencing was performed using the RPT01A-PDTIS sequencing platform, Fiocruz-RJ (ABI 3730XL Applied Biosystem), with the Big Dye Terminator v3.1 Cycle Sequencing Ready Reaction kit (Applied Biosystems, CA, USA). The electropherograms were analysed using the Phred program, and the regions with good sequence resolution were submitted to assembly using the CAP3 program, removing the vector sequence using the NCBIVecScreen program (http://www.ncbi.nlm.nih.gov/VecScreen/VecScreen.html). The sequences were compared with those available in the BLASTnucleotide database (http://blast.ncbi.nlm.nih.gov/Blast.cgi) using the BLASTN algorithm.

## RESULTS AND DISCUSSION

Between May 2017 and July 2018, a total of 1,186 specimens were collected, and, from these, 176 pools of sand fly specimens were isolated to be used for the DNA research of *Leishmania* spp. Among the analysed species, *Ny. neivai* was the most abundant (88.62%), followed by *Pintomyia fischeri* (9.2%) and *Mg. migonei* (1.18%). *Brumptomyia troglodytes* and *Expapillata firmatoi* together totalised 1.01% of the total number of females analysed. There was a remarkable increase in *Ny. neivai* in September and February, which were the warmest and least humid months of the experimental period. This species was observed in animal shelters and indoors, proving its adaptation to modified environments.

Three pools of *Ny. neivai* tested positive for *Leishmania* spp. DNA by PCR. Sequencing confirmed two as *L. braziliensis* and one as *L. infantum*. In the Vale do Ribeira, an endemic area for ATL, the detection of *Ny. neivai* specimens with *L. braziliensis* DNA further supports the presence of this parasite in the region. It indicates that this species may play a central role in transmitting the parasite. Several factors demonstrate the possible vectorial role of *Ny. neivai* for the etiologic agent of ATL in southern Brazil, such as the very low density of other suspected vectors for *L. braziliensis* ^15^ ^,^ ^16^ and the high population density of *Ny. neivai*. Other factors include the constant monthly frequency of this sand fly, combined with a marked seasonality and a clear adaptation to anthropic environments, which present environmental conditions favourable to the persistence of the enzootic cycle of *L. braziliensis*.

The discovery of *L. infantum* DNA introduces a novel aspect to this region. Previously, the circulation of this parasite had only been reported in the municipality of Foz do Iguaçu.^11^ Vector species are geographically distributed in their respective transmission cycles, adapted to the abiotic factors of that habitat. However, as external interactions such as anthropic, climatic, and spatial actions start to act in that originally preserved environment, the distribution of these species can narrow the relationship between the etiological agent and other vectors that are not part of their natural cycle.^17^ Even though the determination of vector competence can only be described after observing the protozoa inside the vector’s gut, the presence of *Leishmania* spp. in sand flies that are not incriminated as vectors is extremely important.^14^

The combination of these factors, along with the findings detailed in our study, serves as a warning regarding the spread of VL in the state. This is particularly significant as *Ny. neivai* has previously been linked to the transmission of *L. infantum* in the Southeast and Southern regions of Brazil. The first report of natural infection of the species by *L. infantum* was described by Saraiva et al.^18^ in an area with no registered cases of VL, in the State of Minas Gerais. In the second report conducted by Dias et al.^4^ in the municipality of Florianópolis, where 11 species of sand flies were collected, with three testing positive for *Leishmania* sp. However, the confirmation of *L. infantum* infection was exclusively observed in *Ny. neivai*.

The *Ny. neivai*’s positivity for *L. infantum* DNA is described in the first report in the State of Paraná, in a municipality that does not have *Lu. longipalpis* in its sand fly fauna, nor cases of VL. In addition, other vectors suspected of transmitting *L. infantum*, *Pi. fischeri* ^3^ and *Mg. migonei* ^2^ ^,^ ^19^ were collected in our study, but without positive pools for *Leishmania* DNA.

Our findings indicate the circulation of *L. infantum* in an area traditionally considered free from VL and reinforce the importance of entomological and health surveillance in this region.

## References

[1] OPAS (2022). Leishmanioses: informe epidemiológico das Américas. https://www.paho.org/pt/documentos/leishmanioses-informe-epidemiologico-das-americas-no-11-dezembro-2022.

[2] Carvalho MR, Valença HF, Silva FJ, Pita-Pereira D, Araújo Pereira T, Britto C (2010). Natural Leishmania infantum infection in Migonemyia migonei (França, 1920) (Diptera Psychodidae: Phlebotominae) the putative vector of visceral leishmaniasis in Pernambuco State, Brazil. Acta Trop.

[3] Galvis-Ovallos F, Silva MD, Bispo GB, Oliveira AG, Neto JR, Malafronte RD (2017). Canine visceral leishmaniasis in the metropolitan area of São Paulo Pintomyia fischeri as potential vector of Leishmania infantum. Parasite.

[4] Dias ES, Michalsky ÉM, Nascimento JC, Castro Ferreira E, Lopes JV, Fortes-Dias CL (2013). Detection of Leishmania infantum, the etiological agent of visceral leishmaniasis, in Lutzomyia neivai, a putative vector of cutaneous leishmaniasis. J Vector Ecol.

[5] Lidani KCF, Andrade FAA, Tizzot MRPA, Costa-Ribeiro MCV, Beltrame MH, Messias-Reason IJ (2017). Visceral leishmaniasis and natural infection rates of Leishmania in Lutzomyia longipalpis in Latin America. Int Open.

[6] Silva AM, Camargo N, Santos DR, Massafera R, Ferreira AC, Postai C (2008). Diversidade, distribuição e abundância de flebotomíneos (Diptera Psychodidae) no Paraná. Neotrop Entomol.

[7] Santos BA, Reinhold-Castro KR, Cristovão EC, Silveira TG, Teodoro U (2016). Sand flies on Paraná River islands and natural infection of Nyssomyia neivai by Leishmania in Southern Brazil. J Vector Ecol.

[8] Pita-Pereira D, Souza GD, Zwetsch A, Alves CR, Britto C, Rangel EF (2009). First report of Lutzomyia (Nyssomyia) neivai (Diptera Psychodidae: Phlebotominae) naturally infected by Leishmania (Viannia) braziliensis in a periurban area of South Brazil using a Multiplex Polymerase Chain Reaction Assay. Am J Trop Med Hyg.

[9] Rangel EF, Lainson R (2009). Proven and putative vectors of American cutaneous leishmaniasis in Brazil aspects of their biology and vectorial competence. Mem Inst Oswaldo Cruz.

[10] MS - Ministério da Saúde (2021). Boletim Epidemiológico: doenças tropicais negligenciadas. https://www.gov.br/saude/pt-br/centrais-de-conteudo/publicacoes/boletins/boletins-epidemiologicos/especiais/2021/boletim_especial_doencas_negligenciadas.pdf.

[11] Thomaz-Soccol V, Gonçalves AL, Piechnik CA, Baggio RA, Boeger WA, Buchman TL (2018). Hidden danger unexpected scenario in the vector-parasite dynamics of leishmaniasis in the Brazil side of triple border (Argentina, Brazil and Paraguay). PLoS Negl Trop Dis.

[12] Galati EAB, Rangel EF, Lainson R, orgs (2003). Flebotomíneos do Brasil.

[13] Marcondes CB (2007). A proposal of generic and subgeneric abbreviations for phlebotomine sandflies (Diptera Psychodidae, Phlebotominae). Entomol News.

[14] Pita-Pereira D, Alves CR, Souza MB, Brazil RP, Bertho AL, Barbosa AF (2005). Identification of naturally infected Lutzomyia intermedia and Lutzomyia migonei with Leishmania (Viannia) braziliensis in Rio de Janeiro (Brazil) revealed by a PCR multiplex non-isotopic hybridization assay. Trans R Soc Trop Med Hyg.

[15] Rangel EF, Lainson R (2003). Flebotomíneos do Brasil.

[16] Rangel EF, Lainson R, Carvalho BM, Costa SM, Shaw JJ, Rangel EF, Shaw JJ (2018). Brazilian sand flies: biology, taxonomy, medical importance and control.

[17] Pearson RG, Dawson TP (2003). Predicting the impacts of climate change on the distribution of species are bioclimate envelope models useful?. Glob Ecol Biogeogr.

[18] Saraiva L, Carvalho GML, Gontijo CMF, Quaresma PF, Lima ACVMR, Falcão AL (2009). Natural infection of Lutzomyia neivai and Lutzomyia sallesi (Diptera Psychodidae) by Leishmania infantum chagasi in Brazil. J Med Entomol.

[19] Guimarães VC, Pruzinova K, Sadlova J, Volfova V, Myskova J, Brandão SP (2016). Lutzomyia migonei is a permissive vector competent for Leishmania infantum. Parasit Vectors.

